# Supplementary material for: Quantifying electrostatic control of docking and binding energetics in functional Cx36 gap junctions
Source: Commun Biol. 2026 Jun 11;9:815. doi: 10.1038/s42003-026-10464-w (PMC13269782; doi:10.1038/s42003-026-10464-w)
Supplement: Supplementary file 2 — Description of additional Supplementary File [file 42003_2026_10464_MOESM2_ESM.pdf]

Description of Additional Supplementary Files

File name: Supplementary_Data_1

Description:

This is an Excel file with 11 sheets of different data. Here are the details:

Sheet name	Descriptions
Gj_for_Fig_1-5_and_Fig_S3	coupling conductance (Gj) for different GJ combinations described in the first row
Fig_1B	changes in electrostatics (ΔΔΨ) and the changes in binding free energy (ΔΔG)
Fig_2E	changes in electrostatic energy (ΔΔΨ) of each GJ combination and predicted ESI numbers
%GJ_Plaques_for_Fig_3E	count (and %) of cell-cell interfaces showing morphological GJ plaques
Fig_4C	changes in electrostatic energy (ΔΔΨ) of each GJ combination and their functional status
Fig_4D_and_Fig_S4B	changes in electrostatic energy (ΔΔΨ) and changes in binding free energy (ΔΔG) of each GJ combination and predicted ESI numbers
Fig_S1	changes in binding free energy (ΔΔG) and individual contributing energy components
Fig_S4A	changes in binding free energy (ΔΔG) and individual contributing energy components of each GJ combination and their functional status
Fig_S5A_and_Fig_S5B	changes in electrostatic energy (ΔΔΨ) and changes in binding free energy (ΔΔG) of each GJ combination and predicted ESI numbers

Fig_S5C	changes in binding free energy (ΔΔG) and individual contributing energy components of each GJ combination and their functional status
Gj_for_Fig_S6	coupling conductance (Gj) for different GJ combinations described in the first row
