# Supplementary material for: Quantifying electrostatic control of docking and binding energetics in functional Cx36 gap junctions
Source: Commun Biol. 2026 Jun 11;9:815. doi: 10.1038/s42003-026-10464-w (PMC13269782; doi:10.1038/s42003-026-10464-w)
Supplement: Supplementary file 4 — Reporting summary [file 42003_2026_10464_MOESM4_ESM.pdf]

nature portfolio

Corresponding author(s): Haiqing Zhao and Donglin Bai

Last updated by author(s): May 29, 2026

Reporting Summary

Nature Portfolio wishes to improve the reproducibility of the work that we publish. This form provides structure for consistency and transparency in reporting. For further information on Nature Portfolio policies, see our Editorial Policies and the Editorial Policy Checklist.

Statistics

For all statistical analyses, confirm that the following items are present in the figure legend, table legend, main text, or Methods section.

n/a	Confirmed	
☐	☒	The exact sample size (*n*) for each experimental group/condition, given as a discrete number and unit of measurement
☐	☒	A statement on whether measurements were taken from distinct samples or whether the same sample was measured repeatedly
☐	☒	The statistical test(s) used AND whether they are one- or two-sided<br>*Only common tests should be described solely by name; describe more complex techniques in the Methods section.*
☒	☐	A description of all covariates tested
☐	☒	A description of any assumptions or corrections, such as tests of normality and adjustment for multiple comparisons
☐	☒	A full description of the statistical parameters including central tendency (e.g. means) or other basic estimates (e.g. regression coefficient) AND variation (e.g. standard deviation) or associated estimates of uncertainty (e.g. confidence intervals)
☐	☒	For null hypothesis testing, the test statistic (e.g. *F*, *t*, *r*) with confidence intervals, effect sizes, degrees of freedom and *P* value noted<br>*Give P values as exact values whenever suitable.*
☒	☐	For Bayesian analysis, information on the choice of priors and Markov chain Monte Carlo settings
☒	☐	For hierarchical and complex designs, identification of the appropriate level for tests and full reporting of outcomes
☒	☐	Estimates of effect sizes (e.g. Cohen's *d*, Pearson's *r*), indicating how they were calculated

Our web collection on statistics for biologists contains articles on many of the points above.

Software and code

Policy information about availability of computer code

Data collection	In this manuscript, we utilized FoldX to compute mutation effects on various terms of energies contributing to the change of free energy. FoldX is an empirical force field originally developed to predict the effect of mutations on protein stability and interaction energies, utilizing a rigid-backbone approximation and detailed side chain rotamer libraries. The program is available at https://foldxsuite.crg.eu/
Data analysis	All computational analysis scripts, datasets, and figure source files generated in this study are available at GitHub (https://github.com/zhaolabutmb/Cx36_Channel_Electrostatics), or Zenodo at (https://zenodo.org/records/20303481).

For manuscripts utilizing custom algorithms or software that are central to the research but not yet described in published literature, software must be made available to editors and reviewers. We strongly encourage code deposition in a community repository (e.g. GitHub). See the Nature Portfolio guidelines for submitting code & software for further information.

Data

Policy information about availability of data

All manuscripts must include a data availability statement. This statement should provide the following information, where applicable:

- Accession codes, unique identifiers, or web links for publicly available datasets
- A description of any restrictions on data availability
- For clinical datasets or third party data, please ensure that the statement adheres to our policy

Numerical source data underlying patch clamp, computational data, and percentage of morphological GJ plaque figures are provided in Supplementary Data 1. Raw patch clamp recordings are available upon reasonable request to the corresponding author.

Research involving human participants, their data, or biological material

Policy information about studies with human participants or human data. See also policy information about sex, gender (identity/presentation), and sexual orientation and race, ethnicity and racism.

Reporting on sex and gender	*Use the terms sex (biological attribute) and gender (shaped by social and cultural circumstances) carefully in order to avoid confusing both terms. Indicate if findings apply to only one sex or gender; describe whether sex and gender were considered in study design; whether sex and/or gender was determined based on self-reporting or assigned and methods used. Provide in the source data disaggregated sex and gender data, where this information has been collected, and if consent has been obtained for sharing of individual-level data; provide overall numbers in this Reporting Summary. Please state if this information has not been collected. Report sex- and gender-based analyses where performed, justify reasons for lack of sex- and gender-based analysis.*
Reporting on race, ethnicity, or other socially relevant groupings	*Please specify the socially constructed or socially relevant categorization variable(s) used in your manuscript and explain why they were used. Please note that such variables should not be used as proxies for other socially constructed/relevant variables (for example, race or ethnicity should not be used as a proxy for socioeconomic status). Provide clear definitions of the relevant terms used, how they were provided (by the participants/respondents, the researchers, or third parties), and the method(s) used to classify people into the different categories (e.g. self-report, census or administrative data, social media data, etc.) Please provide details about how you controlled for confounding variables in your analyses.*
Population characteristics	*Describe the covariate-relevant population characteristics of the human research participants (e.g. age, genotypic information, past and current diagnosis and treatment categories). If you filled out the behavioural & social sciences study design questions and have nothing to add here, write "See above."*
Recruitment	*Describe how participants were recruited. Outline any potential self-selection bias or other biases that may be present and how these are likely to impact results.*
Ethics oversight	*Identify the organization(s) that approved the study protocol.*

Note that full information on the approval of the study protocol must also be provided in the manuscript.

Field-specific reporting

Please select the one below that is the best fit for your research. If you are not sure, read the appropriate sections before making your selection.

☒ Life sciences ☐ Behavioural & social sciences ☐ Ecological, evolutionary & environmental sciences

For a reference copy of the document with all sections, see nature.com/documents/nr-reporting-summary-flat.pdf

Life sciences study design

All studies must disclose on these points even when the disclosure is negative.

Sample size	For patch clamp and visualization of morphological gap junction plaque data, no sample size calculation was performed. Sample sizes were determined based on feasibility of the technique, and consistency with field standards.
Data exclusions	For patch clamp data, cell pairs that had a junctional conductance greater than 50 nS were excluded because it is impossible to differentiate the gap junction-coupled cell pairs from "apparently highly coupled cell pairs" due to cytoplasmic bridge. Additionally, patch clamp recordings with input resistance under 200 MΩ were excluded from the results due to possible poor seal quality and associated voltage clamp errors.
Replication	Patch clamp data for each group was repeated for 3 or more independent transfections, and 12 or more individual cell pairs. Visualization of morphological gap junction plaque data was repeated for 5 independent transfections, where a minimum of 50 cell-to-cell interfaces for each experimental group were evaluated per transfection. Visualization of Cx31.3-GFP, Cx43-GFP, Cx45-GFP, and GFP localization in HEK293 cells was repeated for 3 independent transfections and similar results were obtained.
Randomization	For patch clamp experiments, isolated cell pairs expressing the connexin(s) of interest with fluorescent protein reporters were randomly

Randomization	selected. For visualization of morphological gap junction plaque experiments, the first 50 or more cell-to-cell interfaces in positively expressed cells on the coverslip were selected.
Blinding	Partial data for dual patch clamp and morphological GJ plaque visualization experiments were collected blindly. Due to lack of manpower, the majority of experiments were prepared (transfection and coverslip preparation) and patched by the same investigator, thus blinding was not possible. For similar reasons, data analysis could not be performed blinded. Similarly for the subcellular localization of GFP-tagged Cx31.3, Cx43, and Cx45, blinding was not possible.

Reporting for specific materials, systems and methods

We require information from authors about some types of materials, experimental systems and methods used in many studies. Here, indicate whether each material, system or method listed is relevant to your study. If you are not sure if a list item applies to your research, read the appropriate section before selecting a response.

Materials & experimental systems

n/a	Involved in the study
☒	☐ Antibodies
☐	☒ Eukaryotic cell lines
☒	☐ Palaeontology and archaeology
☒	☐ Animals and other organisms
☒	☐ Clinical data
☒	☐ Dual use research of concern
☒	☐ Plants

Methods

n/a	Involved in the study
☒	☐ ChIP-seq
☒	☐ Flow cytometry
☒	☐ MRI-based neuroimaging

Eukaryotic cell lines

Policy information about cell lines and Sex and Gender in Research

Cell line source(s)	A version of HEK293 cells (AD-293 cells) were obtained from Agilent (catalog part# 240085). Genes encoding Cx43 and Cx45 were knocked out sequentially to generate a connexin-deficient cell line. HEK293 cells are derived from human embryonic kidney cells isolated from a female fetus.
Authentication	No used cell lines were authenticated
Mycoplasma contamination	HEK293 cells used tested negative for mycoplasma.
Commonly misidentified lines (See ICLAC register)	*Name any commonly misidentified cell lines used in the study and provide a rationale for their use.*

Plants

Seed stocks	*Report on the source of all seed stocks or other plant material used. If applicable, state the seed stock centre and catalogue number. If plant specimens were collected from the field, describe the collection location, date and sampling procedures.*
Novel plant genotypes	*Describe the methods by which all novel plant genotypes were produced. This includes those generated by transgenic approaches, gene editing, chemical/radiation-based mutagenesis and hybridization. For transgenic lines, describe the transformation method, the number of independent lines analyzed and the generation upon which experiments were performed. For gene-edited lines, describe the editor used, the endogenous sequence targeted for editing, the targeting guide RNA sequence (if applicable) and how the editor was applied.*
Authentication	*Describe any authentication procedures for each seed stock used or novel genotype generated. Describe any experiments used to assess the effect of a mutation and, where applicable, how potential secondary effects (e.g. second site T-DNA insertions, mosiacism, off-target gene editing) were examined.*
